# Supplementary material for: Rapid turnover of life-cycle-related genes in the brown algae
Source: Genome Biol. 2019 Feb 14;20:35. doi: 10.1186/s13059-019-1630-6 (PMC6374913; doi:10.1186/s13059-019-1630-6)
Supplement: Supplementary file 3 — Protein alignments, corresponding Gblocks html file and CDS sequences for the lineage conserved gametophyte-biased genes. (ZIP 2109 kb) [file 13059_2019_1630_MOESM3_ESM.zip › Con_GA_alignments/Ec-06_006930.aa_ali.fasta-gb.htm]

Ec-06\_006930.aa.fasta.trans.aa\_ali.fasta


## Gblocks 0.91b Results

Processed file: **Ec-06\_006930.aa.fasta.trans.aa\_ali.fasta**  
Number of sequences: **2**  
Alignment assumed to be: **Protein**  
New number of positions: **438** (selected positions are underlined in blue)

```
                         10        20        30        40        50        60
                 =========+=========+=========+=========+=========+=========+
Ec-06_006930     MRQRQQPPGGGGVSGGAAAAGGCDDDDVANDGTTRAVALAAVDEAVRRLLHDAGRAAPSG
MSTRG.29414.1    ------------------------------------------------------------
                                                                             


                         70        80        90       100       110       120
                 =========+=========+=========+=========+=========+=========+
Ec-06_006930     GGGGGGGGGSGGGGGGKNKAVIGVLPKLVPAVTACLEGSTSADGGSSLPPPPAVAAGALG
MSTRG.29414.1    ----------EVGGGGR-QAVISVLPKLVPAITACLEGSTSPDGSSSEPPPQAVAASALG
                                    #########################################


                        130       140       150       160       170       180
                 =========+=========+=========+=========+=========+=========+
Ec-06_006930     VLEALVFLLRSTMRPHVKKVEAATAPWLQGPSDVIRDRAAAVLASLPLCGEAEAWTQAAG
MSTRG.29414.1    ALEALVFLLRSTMRPHVKKVEAATALWLQGPSDVIRDRAAAVLASLPLCGEAEAWTQAAG
                 ############################################################


                        190       200       210       220       230       240
                 =========+=========+=========+=========+=========+=========+
Ec-06_006930     RVALEAHGLLHAAWPDNDVDMGTSDSMSASQLDAALGSGAGLLPPLGG--GAGEGFSGPQ
MSTRG.29414.1    RVALEAHGLLHVAWPDNDVDMASSDSMSTSQVDAALRSGGGLLPPLGAGVGEGEGFSGSQ
                 ###############################################   ##########


                        250       260       270       280       290       300
                 =========+=========+=========+=========+=========+=========+
Ec-06_006930     -----------QGLCHVLELMLTGDSNGRPVAVPVGLILALVERVQRMVSSVVKGQGPQV
MSTRG.29414.1    QVLASLVSRRFKGLCRVLELMLVGDSNGRPVAVPVLLILALVERVQRMASSVVKGHGPQA
                             ################################################


                        310       320       330       340       350       360
                 =========+=========+=========+=========+=========+=========+
Ec-06_006930     FIPVAQGALSHGALSTIRPSLGLAASRLLHALATTEASTVLRIARRFCRALVQGMAKPSD
MSTRG.29414.1    FIPVAQGALSPGALHVIRPALRIATSRVVQALASTEAPTVLRNARRFCRVLVQGLAKPSA
                 ############################################################


                        370       380       390       400       410       420
                 =========+=========+=========+=========+=========+=========+
Ec-06_006930     AGSLRAAGYRAVSRAVLQLGEGAAPYLATPALPAILAEVRSAVAASEPEAAGGGGGAGGD
MSTRG.29414.1    PGSLRAAGYLSVSRAVLQLGEGAAPYLATPALPALLAEARAVVSASDPSASEQGGAGGAT
                 ############################################################


                        430       440       450       460       470       480
                 =========+=========+=========+=========+=========+=========+
Ec-06_006930     SSVEFVAATG-APGVA------AVGGGSKKGMGIEPAMSARKSSQKKQRRSREKEVGRAL
MSTRG.29414.1    DDAPGVAGIGGAGGGAGAGGGSGAAAGGKRSMGIEPAMSARKSSQKKLRRSREKEVGRAL
                 ##########                ##################################


                        490       500       510       520       530       540
                 =========+=========+=========+=========+=========+=========+
Ec-06_006930     AAADAAAAAT--AT-----VASSGGGGGAGRTGAAALAEEQAVCAGLSCLSRVVVCCKGH
MSTRG.29414.1    AAADAAAATVAASNGIVGGVGGGGSGGGAAWTGAAALAEREAACTALSCLSQVVVCCKGH
                 ########           #########################################


                        550       560       570       580       590       600
                 =========+=========+=========+=========+=========+=========+
Ec-06_006930     LPLGGRLAVEEVLHRGLELLSHAGGAAKGSRGDGGGGGGGGGGASGGGRRCARLEDPRVA
MSTRG.29414.1    LPLGGRRAIEEVVYRGLRLVA---------------------------------------
                 ###################                                         


                        610       620       630       640       650       660
                 =========+=========+=========+=========+=========+=========+
Ec-06_006930     REFLGLAHACLMTPLADGTRSGSLSVAIAAFRSFASHPDQRLAAASQQALAGCGAVLYPR
MSTRG.29414.1    ------------------------------------------------------------
                                                                             


                        670       680       690       700       710       720
                 =========+=========+=========+=========+=========+=========+
Ec-06_006930     AAPLHLPHAVTSQARAWGGSSASSTGRGLPMGWGDTLMVAEGESAEEESQEDLEDYQHPG
MSTRG.29414.1    ------------------------------------------------------------
                                                                             


                        730       740       750       760       770       780
                 =========+=========+=========+=========+=========+=========+
Ec-06_006930     RGGGAAGGGGIALTVRAAGLAAEGLTAAAVAGPVVTPPPPPPAAAQTAALEEGGRFPQTV
MSTRG.29414.1    ------------------------------------------------------------
                                                                             


                        790       800       810       820       830       840
                 =========+=========+=========+=========+=========+=========+
Ec-06_006930     PRDASSTSAATAAAASAAAENGDAATTSKSGSGKGKGKGKGRVGGGAAGGGGSGDGGSSA
MSTRG.29414.1    ------------------------------------------------------------
                                                                             


                        850       860       870       880       890       900
                 =========+=========+=========+=========+=========+=========+
Ec-06_006930     GAGAAGTVEEDGHNDNEAEAAAAAAAAAAATAGTRKRPRNDRDGDGDDARIAMDDEEEEE
MSTRG.29414.1    ------------------------------------------------------------
                                                                             


                        910
                 =========+======
Ec-06_006930     EEEEEEEEEEEEYVYX
MSTRG.29414.1    ----------------
```

```
Parameters used
Minimum Number Of Sequences For A Conserved Position: 2
Minimum Number Of Sequences For A Flanking Position: 2
Maximum Number Of Contiguous Nonconserved Positions: 8
Minimum Length Of A Block: 10
Allowed Gap Positions: None
Use Similarity Matrices: Yes
```

```
Flank positions of the 5 selected block(s)
Flanks: [80  227]  [231  240]  [253  430]  [447  488]  [500  559]  

New number of positions in Ec-06_006930.aa.fasta.trans.aa_ali.fasta-gb:  438  (47% of the original 916 positions)
```
